# Epithelial splicing regulatory protein 1 and 2 (ESRP1 and ESRP2) upregulation predicts poor prognosis in prostate cancer

**DOI:** 10.1186/s12885-020-07682-8

**Published:** 2020-12-18

**Authors:** Morton Freytag, Martina Kluth, Elena Bady, Claudia Hube-Magg, Georgia Makrypidi-Fraune, Hans Heinzer, Doris Höflmayer, Sören Weidemann, Ria Uhlig, Hartwig Huland, Markus Graefen, Christian Bernreuther, Corinna Wittmer, Maria Christina Tsourlakis, Sarah Minner, David Dum, Andrea Hinsch, Andreas M. Luebke, Ronald Simon, Guido Sauter, Thorsten Schlomm, Katharina Möller

**Affiliations:** 1 Institute of Pathology, University Medical Center Hamburg-Eppendorf, Martinistr. 52, 20246 Hamburg, Germany; 2 Martini-Clinic, Prostate Cancer Center, University Medical Center Hamburg-Eppendorf, Hamburg, Germany; 3 Department of Urology, Charité - Universitätsmedizin Berlin, Berlin, Germany

**Keywords:** ESRP1, ESRP2, Prostate cancer, Tissue micro array, Prognosis

## Abstract

**Background:**

Epithelial splicing regulatory protein 1 (ESRP1) and 2 (ESRP2) regulate alternative splicing events of various pre-mRNAs. Some of these targets play a role in cancer-associated processes, including cytoskeleton reorganization and DNA-repair processes. This study was undertaken to estimate the impact of ESRP1 and ESRP2 alterations on prostate cancer patient prognosis.

**Methods:**

A tissue microarray made from 17,747 individual cancer samples with comprehensive, pathological, clinical and molecular data was analyzed by immunohistochemistry for ESRP1 and ESRP2.

**Results:**

Nuclear staining for ESRP1 was seen in 38.6% (36.0% low, 2.6% high) of 12,140 interpretable cancers and in 41.9% (36.4% low, 5.3% high) of 12,962 interpretable cancers for ESRP2. Nuclear protein expression was linked to advanced tumor stage, high Gleason score, presence of lymph node metastasis, early biochemical recurrence, and ERG-positive cancers (*p* < 0.0001 each). Expression of ESRPs was significantly linked to 11 (ESRP1)/9 (ESRP2) of 11 analyzed deletions in all cancers and to 8 (ESRP1)/9 (ESRP2) of 11 deletions in ERG-negative cancers portending a link to genomic instability. Combined ESRPs expression analysis suggested an additive effect and showed the worst prognosis for cancers with high ESRP1 and ESRP2 expression. Multivariate analyses revealed that the prognostic impact of ESRP1, ESRP2 and combined ESRP1/ESRP2 expression was independent of all established pre- and postoperative prognostic features.

**Conclusions:**

Our data show a striking link between nuclear ESRP expression and adverse features in prostate cancer and identifies expression of ESRP1 and/or ESRP2 as independent prognostic markers with a potential for routine application.

## Background

Prostate cancer was responsible for approximately 358,000 cancer related deaths with 1,270,000 newly diagnosed cases in 2018 and is the most common cancer in Western societies [1]. The clinical course is highly variable ranging from highly aggressive to harmless with no need for therapy. Until today, established prognostic parameters include clinical stage, serum level of prostate specific antigen (PSA), tumor extent and preoperative Gleason grade. Better (molecular) markers are needed to predict tumor behavior and identify patients with no need for therapy.

Epithelial splicing regulatory proteins (ESRP1 and ESRP2) are members of the heterogeneous nuclear ribonucleoprotein (hnRNP) family of RNA binding proteins that plays a role in the regulation of alternative splicing events of pre-mRNAs [2]. In situ hybridization of whole tissue sections from mice showed specific epithelial expression of ESRPs in diverse tissues and organs suggesting that ESRPs maintain epithelial phenotype development during epithelial-mesenchymal-transition (EMT) [3–5]. Some ESRP-regulated mRNA splice variants are involved in regulating cytoskeleton reorganization, cell adhesion, and DNA-repair processes [5–7]. ESRP1 and ESRP2 share similar structural features with well conserved RNA-recognition motifs and exhibit at least some functional redundancy [2].

Overexpression of ESRP1 and/or ESRP2 has been described in various malignant tumors, such as pancreatic ductal adenocarcinoma, oral squamous carcinoma, ovarian cancer, and luminal-type breast cancer [8–12]. In prostate cancer, a meta-analysis reported significant up-regulation of ESRP1 and ESRP2 mRNAs in 719 prostate cancers from 11 previous studies including normal and malignant prostate tissues [13]. There is conflicting data on the prognostic significance of ESRP expression. While some studies found a positive impact on prognosis as in pancreatic ductal adenocarcinoma and colorectal carcinoma [12, 14], others linked elevated ESRP expression to poor patient prognosis in breast cancer [10] and prostate cancer [15]. Our group recently identified ESRP1 to be significantly overexpressed in prostate cancer using an RNA expression screening approach and found that high ESRP1 expression detected by immunohistochemistry (IHC) was an independent predictor of a shorter time to biochemical recurrence [16]. Other prostate cancer studies on ESRP1 or ESRP2 IHC expression are so far lacking.

To determine whether ESRP2 has a similar prognostic effect compared to ESRP1 and whether a combined analysis of multiple ESRP family members would result in even better prognostic information, immunohistochemical analysis was performed on a tissue microarray (TMA) containing more than 17,000 prostate cancers with long-term follow-up data.

## Methods

### Patients

Radical prostatectomies of 17,747 patients treated at the Department of Urology and the Martini Clinic at the University Medical Center Hamburg-Eppendorf between 1992 and 2014 were available. All prostatectomies were processed according to a standardized procedure, including a complete embedding of the entire prostate for histological analysis [17]. Histopathological parameters were available from the patients’ records, including Gleason grade, pathological tumor stage (pT), presents of lymph node metastasis (pN), and presents of tumor cells in the resection margin (R). In addition to the classical Gleason score categories, “quantitative” Gleason grading was done as described before [18]. In short, for every individual prostatectomy specimen, the percentage of Gleason 4 patterns in neoplastic tissues were estimated and the group of Gleason score 3 + 4 and 4 + 3 cancers were subdivided for practical use in 8 subgroups as follows: 3 + 4 with ≤5% Gleason 4, 3 + 4 with 6–10% Gleason 4, 3 + 4 with 11–20% Gleason 4, 3 + 4 with 21–30% Gleason 4, 3 + 4 with 31–49% Gleason 4, 4 + 3 with 50–60% Gleason 4, 4 + 3 with 61–80% Gleason 4 and 4 + 3 with > 80% Gleason 4. In addition, two subgroups were defined by the presence of a tertiary Gleason 5 pattern (3 + 4 Tert. 5 and 4 + 3 Tert. 5). For 14,464 patients, follow-up data were available (median: 48.0 months; range: 1 to 276 months; Table 1). PSA recurrence was defined as the time point at which the postoperative PSA level rose to at least 0.2 ng/ml. The production of TMAs has already been described in detail [19]. In brief, from each individual patient a 0.6 mm core was removed from a cancer containing tissue block. The molecular database associated with the TMA include IHC results on ERG expression in 13,089 [20] and ESRP1 in 12,140 tumors [16], and fluorescence in situ hybridization (FISH) results on ERG breakage in 7225 (expanded from [20]) as well as on deletion status of 3p13 (*FOXP1*) in 7201 (expanded from [21]), 5q21 (*CHD1*) in 8074 (expanded from [22]), 6q15 (*MAP 3 K7*) in 6171 (expanded from [23]), 8p21 (*NKX3.1*) in 7001 [24], *PTEN* (10q23) in 6803 (expanded from [25]), 12p13 (*CDKN1B*) in 6187 [26], 12q24 (*NCOR2*) in 7435 [20], 13q14 (*ENOX1*) in 7499 [27], 16q23 *(WWOX*) in 3928 [28], 17p13 (*TP53*) in 8307 [29], and 18q24 in 7032 [30] cancers.

**Table 1:** Study cohort (*n* = 17,747)

	No. of patients (%)	
	Study cohort on TMA	Biochemical relapse among categories
	(n = 17,747)	
**Follow-up (mo)**		
n	14,464 (81.5%)	3612 (25%)
Mean	56.3	–
Median	48	–
Age (y)		
≤ 50	433 (2.4%)	66 (15.2%)
51–59	4341 (24.5%)	839 (19.3%)
60–69	9977 (56.4%)	2073 (20.8%)
≥ 70	2936 (16.6%)	634 (21.6%)
**Pretreatment PSA (ng/ml)**		
< 4	2225 (12.6%)	313 (14.1%)
4–10	10,520 (59.6%)	1696 (16.1%)
10–20	3662 (20.8%)	1043 (28.5%)
> 20	1231 (7%)	545 (44.3%)
**pT stage (AJCC 2002)**		
pT2	11,518 (65.2%)	1212 (10.5%)
pT3a	3842 (21.7%)	1121 (29.2%)
pT3b	2233 (12.6%)	1213 (54.3%)
pT4	85 (0.5%)	63 (74.1%)
**Gleason grade**		
≤ 3 + 3	3570 (20.3%)	264 (7.4%)
3 + 4	9336 (53%)	1436 (15.4%)
3 + 4 Tert.5	798 (4.5%)	165 (20.7%)
4 + 3	1733 (9.8%)	683 (39.4%)
4 + 3 Tert.5	1187 (6.7%)	487 (41%)
≥ 4 + 4	999 (5.7%)	531 (53.2%)
**pN stage**		
pN0	10,636 (89.4%)	2243 (21.1%)
pN+	1255 (10.6%)	700 (55.8%)
**Surgical margin**		
Negative	14,297 (80.8%)	2307 (16.1%)
Positive	3388 (19.2%)	1304 (38.5%)

NOTE: Percent in the column “Study cohort on TMA” refers to the fraction of samples across each category. Percent in colum “Biochemical relaps among categories” refers to the fraction of samples with biochemical relaps within each parameter in the different categories. Numbers do not always add up to 17,747 in the different categories because of cases with missing data. Abbreviation: *AJCC* American Joint Committee on Cancer

### Immunohistochemistry

Freshly cut TMA sections were processed in a single run in 1 day. TMAs were deparaffinized and exposed to heat-induced antigen retrieval for 5 min in an autoclave in pH 7.8 Tris-EDTA buffer at 121 °C. Primary antibodies specific against ESRP2 protein (rabbit polyclonal antibody, Sigma-Aldrich, St. Louis, Missouri, USA, HPA0485597; dilution 1:450) and ESRP1 protein (rabbit polyclonal antibody, Sigma Aldrich Germany, cat#HPA023720; dilution 1:450) were incubated for 60 min at 37 °C. The EnVision Kit (Agilent, CA, USA) was used to visualize bound antibody according to the manufacturer’s instructions. ESRP2 staining was seen in the nuclei of prostate epithelial cells and was sometimes accompanied by cytoplasmic staining. Since splicing occurs in the nucleus, we assumed nuclear expression to be biologically relevant and only scored nuclear staining. ESRP2 positive staining was usually seen in all tumor cells (100%). Therefore, the staining intensity was estimated in three categories, i.e. negative (not detectable), low (1–2+) and high (3+) staining. Immunohistochemical ESRP1 data were available from a previous study [16]. To test the combined impact of ESRP1 and ESRP2 expression on prostate cancer prognosis an ESRP1/ESRP2 score was generated as follows: score 0: ESRP1 and ESRP2 negative, score 1: ESRP1 low and ESRP2 negative or ESRP1 negative and ESRP2 low, score 2: ESRP1 and ESRP2 low, score 3: ESRP1 high and ESRP2 negative or ESRP1 negative and ESRP2 high, score 4: ESRP1 high and ESRP2 low or ESRP1 low and ESRP2 high, and score 5: ESRP1 high and ESRP2 high.

### Antibody validation

ESRP1 and ESRP2 antibody specificity was validated in control cell lines with ectopic ESRP1 and ESRP2 protein overexpression (Supplementary Fig. 2). To produce these cells, cDNAs encoding ESRP1/RBM35A (# HG13708-UT, Sino Biological Inc., Wayne PA, USA) and ESRP2 (#HG23639-UT, Sino Biological Inc., Wayne PA, USA) were transformed in competent *Escherichia coli* cells (One ShotTM Top10, TermoFisher Scientifc, Germany), the plasmid DNA was isolated (NucleoBond BAC 100 Kit, #740579, Macherey-Nagel, Düren, Germany) and transfected to cultivated HeLa cells (15 μg/70% confluence/1500 mm dish) using JetPEI DNA Transfection Reagent (Polyplus-transfection, #101-10 N S.A., Illkirch, France). Transfected cells were harvested after 24 h, centrifuged at 1000×g for 5 min, stabilized in agarose, fixed in 4% buffered formalin overnight and embedded in paraffin (FFPE fixation). Non-transfected HeLa cells were cultivated (37 °C and 5% CO_2_) in Dulbecco’s Modified Eagles Medium (DMEM), supplemented with 10% fetal bovine serum (FBS) and 1% penicillin-streptomycin (P/S), harvested and FFPE fixed and served as negative control. For immunohistochemistry, freshly cut section of negative and positive control cell lines were stained as described above.

### Statistics

For statistical calculations the JMP® 14 software (SAS Institute Inc., NC, USA) was used. Chi^2^-tests and contingency tables were used to find associations between ESRP1/ESRP2 and molecular or histopathological parameters. Survival curves were calculated according to Kaplan-Meier. Significant differences between groups were detected by the Log-Rank test. The statistical independence and significance between pathological, molecular and clinical variables was tested by different Cox proportional hazards regression analyses.

## Results

### Technical issues

In our TMA analyses, 12,140 (68.4%) were interpretable for ESRP1 and 12,962 (73.0%) for ESRP2 (out of a total of 17,747 tumor samples). Reasons for non-informative cases (*n* = 5607; 31.6% for ESRP1 and *n* = 4785; 27.0% for ESRP2) included lack of tissue samples or absence of unequivocal cancer tissue in the TMA spot.

### ESRPs expression in normal and cancerous prostate tissues

**Fig. 1:**
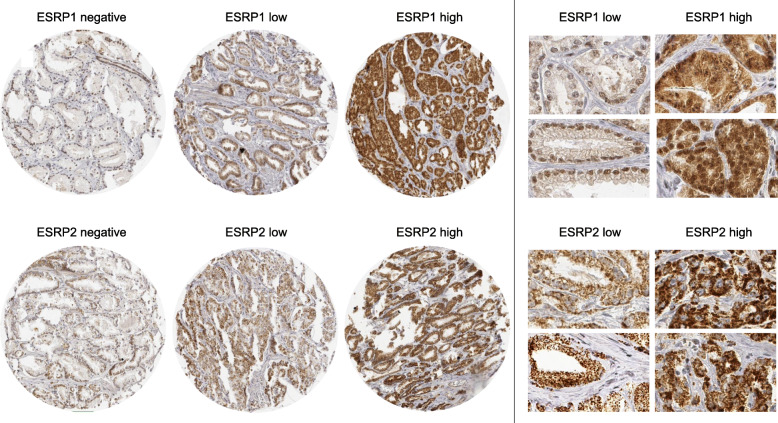
Examples of ESRP1 and ESRP2 cytoplasmic and nuclear immunostaining in prostate cancer. Left side: Immunostaining on the whole TMA spot. Right side: Selected parts of a TMA spot

In normal prostate glands, both ESRP1 and ESRP2 staining in the nucleus was rare and, if present, faint. In prostate cancers, nuclear ESRP1 and ESRP2 staining was more common and also more intense (Fig. 1). For ESRP1, positive nuclear staining was recorded in 4689 (38.6%) of 12,140 interpretable tumors, including 36.0% with low and 2.6% with high staining intensity. For ESRP2, positive nuclear staining was seen in 5393 (41.6%) of 12,962 interpretable cancers. Of these cancers, 36.4% showed low and 5.2% showed high staining intensity. A combined staining for ESRP1 and ESRP2, was detectable in 2503 (21.6%) of 11,597 for ESRP1 and ESRP2 interpretable cancers.

### ESRPs expression and *TMPRSS2*:*ERG* fusion status

**Fig. 2:**
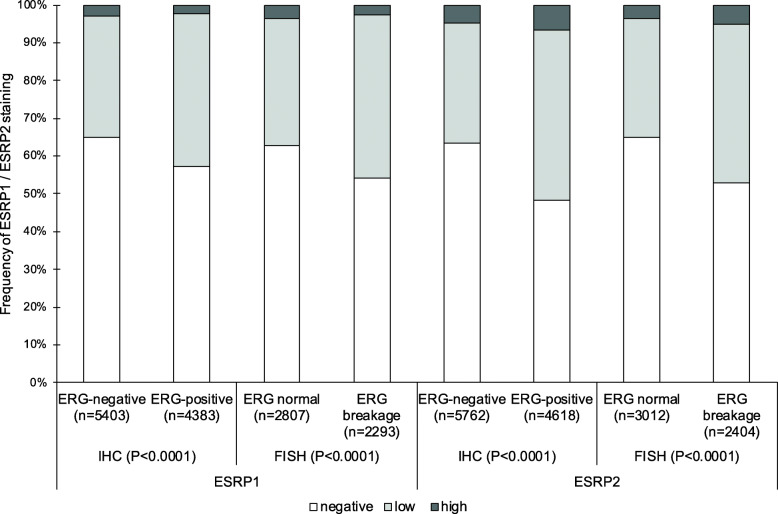
ESRP1 / ESRP2 and *TMPRSS2:ERG* fusion. Contingency tables and the chi^2^-test were performed for ESRP1 on 9786 (immunohistochemistry, IHC) and 5100 (fluorescence in situ hybridization, FISH) analyzable cancers and for ESRP2 on 10,380 (IHC) and 5416 (FISH) analyzable cancers

The *TMPRSS2:ERG* fusion status of the prostate cancers on the TMA has been determined previously by FISH and IHC [20]. The combined data revealed 5100 (ERG status by FISH)/9786 (ERG status by IHC) tumors with evaluable ESRP1 immunostaining and 5416 (ERG status by FISH)/10,380 (ERG status by IHC) tumors with evaluable ESRP2 immunostaining. Data on ERG status determined by FISH as well as IHC were available for 4236 tumors with ESRP1 and 4484 tumors with ESRP2 immunostaining. Identical results for ERG status determined by FISH or IHC were shown in 3850 (90.9%; ESRP1) and in 4073 (90.8%; ESRP2) of these cases. Nuclear staining of both ESRP1 and ESRP2 was linked to *TMPRSS2:ERG* fusion and ERG expression. For example, the fraction of tumors with detectable ESRP1 expression increased from 35.1% in ERG-negative cancers to 42.8% in ERG-positive cancers and with detectable ESRP2 expression from 36.7% in ERG-negative cancers to 51.6% in ERG-positive cancers (Fig. 2).

### ESRPs expression and chromosomal deletions

**Fig. 3:**
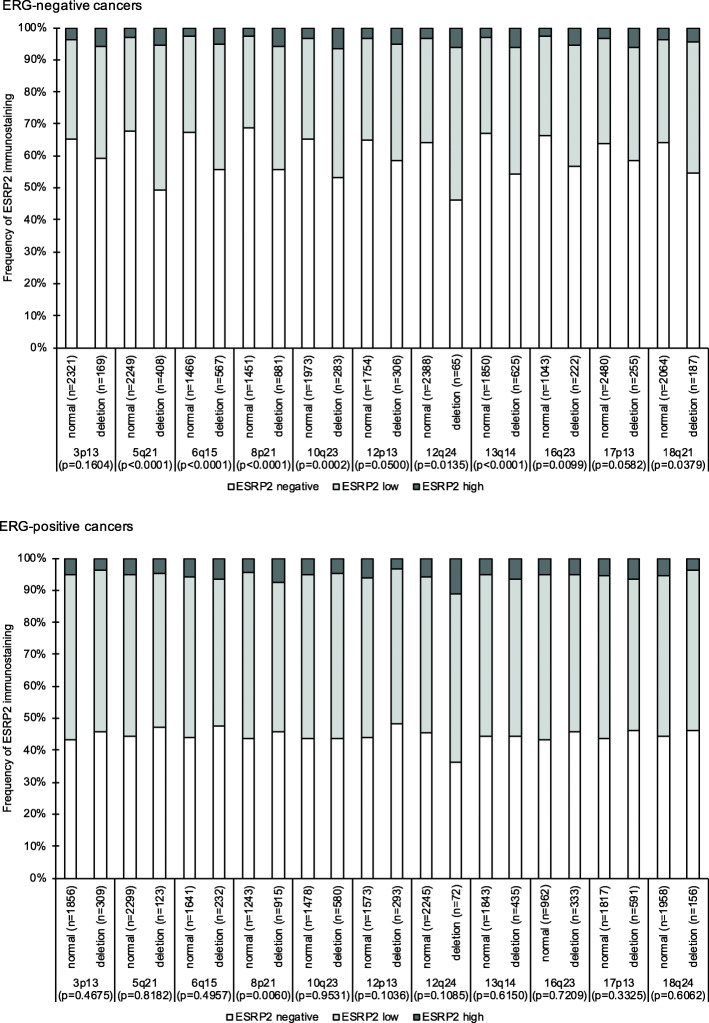
ESRP2 and common chromosomal deletions. For statistical analysis contingency tables and the chi^2^-test were performed

For most of 11 analyzed chromosomal regions, ESRP1 and ESRP2 expression was significantly more common in deleted than in non-deleted cancers in all analyzed cancers (11/11 for ESRP1 and 9/11 for ESRP2, *p* < 0.03 each, data not shown). In ERG-negative cancers these statistically significant associations were retained for ESRP1 in 8 and for ESRP2 in 9 chromosomal regions. In ERG-positive cancers, a statistically significant difference was found for ESRP1 in 5 and for ESRP2 in 1 of the analyzed loci (Fig. 3 and Supplementary Fig. 1).

### ESRPs expression and prostate cancer phenotype and prognosis

**Fig. 4:**
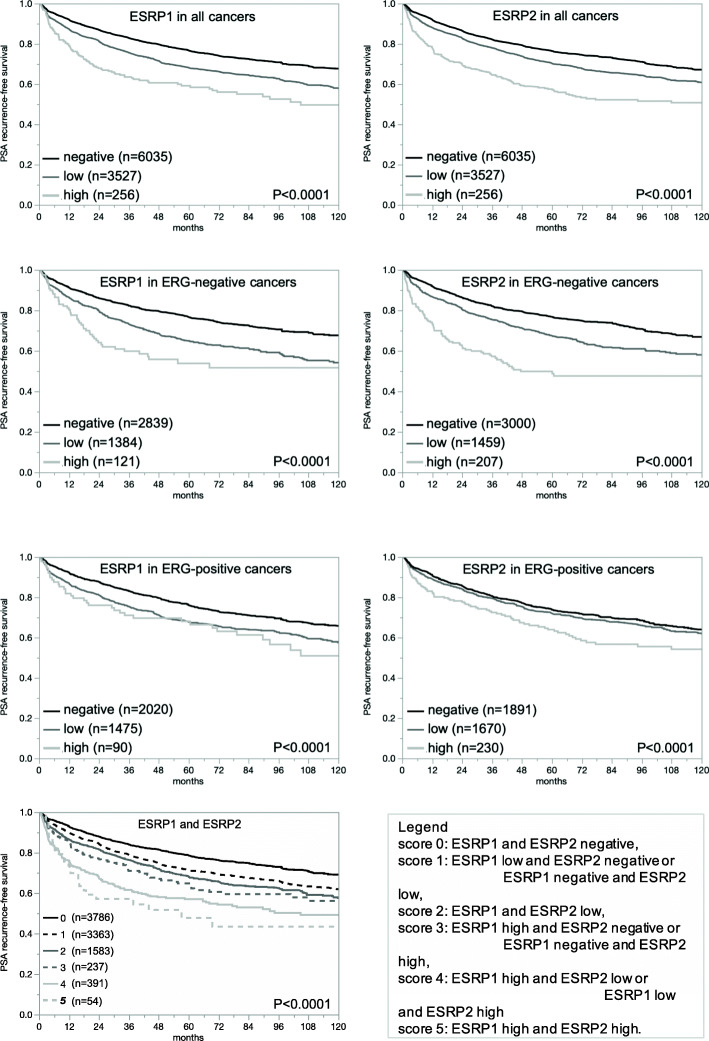
Prognostic relevance of ESRP1 in all cancers (*n* = 9818), ERG-negative cancers (*n* = 4344), and ERG-positive cancers (*n* = 3585), of ESRP2 in all cancers (n = 9818), ERG-negative cancers (*n* = 4666), and ERG-positive cancers (*n* = 3791), and combined ESRP1/ESRP2 status in all cancers (*n* = 9414). Survival curves were calculated according to Kaplan-Meier. The Log-Rank test was applied to detect significant differences between groups

Both, high ESRP1 and high ESRP2 staining were significantly associated with adverse tumor features, including advanced tumor stage, high Gleason grade, presence of lymph node metastasis (*p* < 0.0001 each, Table 2), and high early PSA recurrence (*p* < 0.0001 each; Fig. 4a-b). Most of these associations were also seen in the subsets of ERG-negative and ERG-positive cancers (Fig. 4c-f, Supplementary Tables 1 and 2). The ESRP1/ESRP2 score analysis showed a striking combined impact of combined ESRP1 and ESRP2 expression on prostate cancer prognosis. The higher the score (score 5 = both markers high), the more likely was an early PSA recurrence (Fig. 4g).

**Table 2:** ESRP1 and ESRP2 and prostate cancer phenotype. Contingency tables and the chi^2^-test were performed on 12,140 (ESRP1) and 12,962 (ESRP2) analyzable cancers

	ESRP1 immunostaining					ESRP2 immunostaining				
Parameter	n evaluable	negative (%)	low (%)	high (%)	***p*** value	n evaluable	negative (%)	low (%)	high (%)	***p*** value
**All cancers**	12,140	61.4	36	2.6		12,962	58.4	36.4	5.2	
**Tumor stage**										
pT2	7634	64.32	33.84	1.85	< 0.0001	8163	61.67	34.61	3.72	< 0.0001
pT3a	2759	58.54	38.02	3.44		2950	54.44	38.68	6.88	
pT3b-pT4	1698	52.47	43.05	4.48		1798	50.28	40.49	9.23	
**Gleason grade**										
≤ 3 + 3	2097	70.48	28.47	1.05	< 0.0001	2299	67.33	30.23	2.44	< 0.0001
3 + 4	6529	63.06	35.2	1.75		6906	59.98	36.1	3.92	
3 + 4 Tert.5	592	63.01	33.95	3.04		618	54.85	39.97	5.18	
4 + 3	1214	51.65	42.92	5.44		1291	51.98	39.74	8.29	
4 + 3 Tert.5	895	52.29	43.69	4.02		948	49.05	41.56	9.39	
≥ 4 + 4	725	45.38	46.9	7.72		793	43.51	42.24	14.25	
**Lymph node metastasis**										
N0	7343	59.14	37.98	2.87	< 0.0001	7767	56.43	37.96	5.61	< 0.0001
N+	931	51.34	43.93	4.73		1004	47.91	41.43	10.66

### Multivariate analysis

Four different multivariate analyses were applied to evaluate whether ESRP1 or ESRP2 expression as well as our ESRP1/ESRP2 score is a statistically independent prognostic marker in all prostate cancers and the subset of ERG-negative and ERG-positive cancers (Table 3). Scenario 1 evaluated all parameters available after surgery, including pathological tumor stage (pT), pathological nodal stage (pN), surgical margin status, preoperative PSA value and Gleason grade obtained after evaluation of the entire prostate. In scenario 2, all postoperatively available parameters with exception of nodal status were included. This was because the indication and extent of lymph node dissection is not standardized in the surgical therapy of prostate cancer and more often executed if aggressive cancer is expected based on biopsy results. This may introduce a bias towards high grade cancers in the cohort with available lymph nodes. Two additional scenarios had the purpose to model the preoperative situation to the best possible extent. Scenario 3 included preoperative PSA, clinical tumor stage, and Gleason grade obtained on the prostatectomy specimen. Since a postoperative determination of the Gleason grade is “better” than the preoperatively determined Gleason grade (subjected to sampling errors and consequently under-grading in more than one third of cases), this parameter was replaced by the original preoperative biopsy Gleason grade in Scenario 4. These analyses identified ESRP1 expression, ESRP2 expression as well as our ESRP1/ESRP2 score as independent prognostic parameters in all cancers as well as the subset of ERG-negative and ERG-positive cancers in all four scenarios (*p* ≤ 0.05, Table 3).

**Table 3:** Cox proportional hazards regression analysis in all cancers (*n* = 6412 to 9708), ERG-negative (*n* = 2880 to 4550), and ERG-positive cancers (*n* = 2331 to 3531)

Tumor subset	Scenario	n analyzable	***p*** -value									
			preoperative PSA-Level	pT Stage	cT Stage	Gleason grade prostatectomy	Gleason grade biopsy	pN Stage	R Stage	ESRP1-Expression	ESRP2-Expression	ESRP1/ ESRP2 Score
**all cancers**	1	6412	< 0.0001	< 0.0001	–	< 0.0001	–	< 0.0001	0.0003	0.0014	0.0002	< 0.0001
	2	9708	< 0.0001	< 0.0001		< 0.0001			< 0.0001	0.0006	0.0004	< 0.0001
	3	9565	< 0.0001		< 0.0001	< 0.0001				0.0008	0.0006	0.0001
	4	8133	< 0.0001		< 0.0001		< 0.0001			< 0.0001	< 0.0001	< 0.0001
**ERG-negative cancers**	1	2880	< 0.0001	< 0.0001		< 0.0001		< 0.0001	0.0775	0.0120	0.0017	0.0028
	2	4299	< 0.0001	< 0.0001		< 0.0001			0.0002	0.0079	0.0019	0.0095
	3	4245	< 0.0001		< 0.0001	< 0.0001				0.0050	0.0013	0.0073
	4	4550	< 0.0001		< 0.0001		< 0.0001			< 0.0001	< 0.0001	< 0.0001
**ERG-positive cancers**	1	2331	< 0.0001	< 0.0001		< 0.0001		< 0.0001	0.0003	0.0305	0.0688	0.0008
	2	3531	< 0.0001	< 0.0001		< 0.0001		< 0.0001	< 0.0001	0.0033	0.1063	0.0004
	3	3464	< 0.0001		< 0.0001	< 0.0001				0.0083	0.2992	0.0013
	4	2991	< 0.0001		< 0.0001		< 0.0001			0.0002	0.8306	0.0004

## Discussion

Our study identifies high expression of ESRP1 and ESRP2 as strong and statistically independent prognostic markers in prostate cancer.

Both antibodies used in this study often resulted in some additional cytoplasmatic staining in cells of all types in a TMA spot. Considering, that splicing is confined to the nucleus, we scored only the nuclear staining in this study. Nuclear staining of both ESRPs in normal glands was uncommon and - if present - faint in normal prostatic glands. Positive nuclear ESRP1 and ESRP2 staining was more common in cancers and was recorded in 39 and 42%, respectively, of all analyzable cancers. This is in line with one meta-analysis describing significant upregulation of ESRP1 and ESRP2 mRNA in 719 analyzed prostate cancers from 11 previous studies compared to normal prostate tissue [13]. Increased nuclear ESRP1 and ESRP2 staining in comparison to normal tissues was also found in several other tumor entities, including pancreatic ductal adenocarcinomas [12], oral squamous cell carcinomas [10], ovarian carcinomas [11], and colorectal carcinomas [31]. Overall, these data suggest that transition from normal to neoplastic epithelium may often involve ESRP activation.

The fact that nuclear ESRP1 and ESRP2 overexpression were strongly associated with unfavorable prostate cancer phenotype and poor patient outcome is in line with previous studies describing a link of high ESRPs expression and an unfavorable phenotype in breast [10, 15] and ovarian cancer [11]. In contrast, previous studies have also reported a high ERSP expression to be linked with favorable tumor parameters in pancreatic [12] and colorectal adenocarcinoma [14]. Several functional studies on cell line models have also supported a tumor suppressive rather than an oncogenic role of ESRPs. ESRPs were found to be downregulated after crossing multiple barriers in a PC-3 cell line model for metastasis-building [32]. Breast cancer cell lines with a luminal phenotype (more likely to be associated with a good prognosis) showed higher ESRP expression than cell lines with a basal phenotype (more likely to be associated with a worse prognosis) [9]. ESRP1 knockdown promoted migration and invasion of tumor cells in a model for pancreatic adenocarcinoma [12]. Both ESRP1 and ESRP2 are believed to be responsible for retaining epithelial phenotypes in cancer cells and thus inhibiting EMT [3, 5, 33]. However, other effects of ESRPs may promote tumor progression that outweigh the effect on EMT in certain cell types. Taken together the available data are consistent with variable functional roles of ESRPs depending on the tumor type.

The molecular data that were previously reported for the tumors of this prostate cancer TMA enabled us to investigate the relationship of ESRPs expression with other parameters of interest. For this study, we selected *TMPRSS2:ERG* fusion as it represents the most common genomic alteration in prostate cancer as well as the next most prevalent genomic alterations in prostate cancer which included deletion of 3p, 5q, 6q, 8p, 10q23, 12p, 13q, 16q, 17p, and 18q. *TMPRSS2:ERG* fusions occur in about 50% of prostate cancers, preferably in younger patients [20, 34]. *TMPRSS2:ERG* fusions result in androgen receptor (AR) dependent overexpression of the ETS transcription factor ERG [20, 35]. While the overexpression of ERG itself does not influence prognosis, ERG modulates more than 1600 genes in prostate epithelial cells [34, 36]. In our study, both ESRP1 and ESRP2 were more frequently expressed in ERG-positive than in ERG-negative cancers. This fits well to a recent report demonstrating that ESRP1/2 are AR responsive genes like ERG [13, 37]. Of note, the AR dependency of ESRP1/2 expression connects androgen signaling to alterative splicing. It has been shown that ESRP1/2 activation leads to oncogenic activation of several ESRP1/2 target genes such as MAP 3 K7, mTOR, GSK3ß, RB1, CTNND1, E-Cadherin, and CD44, which drive tumor cell proliferation and EMT, key features of advanced and aggressive cancers [5, 13, 37, 38]. Accordingly, it has been suggested that potential future anti-ESRP1/2 drugs might be particularly effective in combination with androgen deprivation therapy [13, 37].

Elevated expression of ESRP1 and ESRP2 was significantly associated with the vast majority of analyzed chromosomal deletions. This either links ESRPs overexpression to chromosomal instability induced by an increased propensity to undergo DNA double strand breaks or to other mechanisms that are generally connected to cellular dedifferentiation and genetic instability. Since ESRPs are not known to play a direct role in DNA damage response or repair, the second hypothesis might be more likely. This is supported by the broad range of ESRP splicing targets, including many genes with impact on cell cycle control (e.g. RB1), cell adhesion (E-Cadherin, CD44), growth signaling (e.g. FGFR2, EGFR) or chromatin remodeling (CUL4A) [5, 6, 39, 40].

The striking association of ESRP expression with prostate cancer prognosis represents the most notable finding of this study. The fact that the prognostic role of ESRP1, ESRP2 and of the combined ESRP1/ESRP2 score was independent of all established prognostic parameters is suggestive for a possible clinical application of ESRP measurement. Of note, the Gleason score, the strongest preoperatively prognostic parameter suffers from clinically relevant interobserver variability reaching up to 40%, even between experts [41, 42]. Biomarkers are thus needed, that are not only independent of the Gleason score and other established prognostic markers but also show a higher reproducibility. For the future, we expect, that panels of antibodies will assist in the evaluation of prostate cancer aggressiveness. Multicolor immunofluorescence enables the parallel analysis of multiple antibodies and also offers improved quantification. ESRP IHC could become part of a multiparametric prognostic test in the future. The similar prognostic role of ESRP overexpression in ERG-positive and ERG-negative cancers is a distinct advantage for using these proteins in routine diagnostics. Several other prognostic molecular features exert their prognostic role either in ERG-positive [43–45] or in ERG-negative cancers [46–48].

The availability of a large prostate cancer tissue microarray with attached clinical and molecular database is the strength of this study. It allows for highly standardized analysis of multiple markers. However, a drawback of this particular study was that ESRP1 and ESRP2 were not analyzed on consecutive TMA sections so that the results may be affected by possible intratumoral heterogeneity. Analysis of only a single 0.6 mm tissue spot per patient is another limitation of our study. In case of tumor heterogeneity, it cannot be excluded that more tumors are positive for ESRP1/2 than reported in our study. However, studies have shown that large TMAs with a single tissue core are optimally suited to find relevant associations between tumor phenotype and molecular alterations [49–51].

## Conclusions

These results of our study suggest a pivotal role of ESRPs in prostate cancer biology and demonstrate a strong and independent prognostic role of ESRP1 and ESRP2 overexpression.

## Supplementary Information

**Additional file 1 Supplementary Table 1**. ESRP1 and prostate cancer phenotype in ERG-negative (*n* = 4211) and ERG-positive (*n* = 3339) cancers. For statistical analysis contingency tables and the chi^2^-test were performed. **Supplementary Table 2**. ESRP2 and prostate cancer phenotype in ERG-negative (*n* = 4555) and ERG-positive (*n* = 3508) cancers. For statistical analysis contingency tables and the chi^2^-test were performed. **Supplementary Figure 1**. ESRP1 and common chromosomal deletions. For statistical analysis contingency tables and the chi^2^-test were performed. **Supplementary Figure 2**. Validation of ESRP1 and ESRP2 antibodies. ESRP1: Strong staining in ESRP1 overexpressing Hela cells (positive control) and no staining in HeLa wildtype cells (negative control). ESRP2: Strong staining in ESRP2 overexpressing Hela cells (positive control) and no staining in Hela wildtype cells (negative control).

## Data Availability

All data generated or analyzed during this study are included in this published article [and its supplementary information files].

## Abbreviations

ESRP: Epithelial splicing regulatory protein
PSA: Prostate specific antigen
hnRNP: Heterogeneous nuclear ribonucleoprotein
EMT: Epithelial-mesenchymal-transition
IHC: Immunohistochemistry
TMA: Tissue microarray
ERG: V-Ets Avian Erythroblastosis Virus E26 Oncogene Related
TMPRSS2:ERG: Transmembrane protease, serine 2: ETS-related gene fusion
