# Supplementary material for: Epithelial splicing regulatory protein 1 and 2 (ESRP1 and ESRP2) upregulation predicts poor prognosis in prostate cancer
Source: BMC Cancer. 2020 Dec 18;20:1220. doi: 10.1186/s12885-020-07682-8 (PMC7749503; doi:10.1186/s12885-020-07682-8)
Supplement: Supplementary file 1 — Additional file 1 Supplementary Table 1. ESRP1 and prostate cancer phenotype in ERG-negative (n = 4211) and ERG-positive (n = 3339) cancers. For statistical analysis contingency tables and the chi2-test were performed. Supplementary Table 2. ESRP2 and prostate cancer phenotype in ERG-negative (n = 4555) and ERG-positive (n = 3508) cancers. For statistical analysis contingency tables and the chi2-test were performed. Supplementary Figure 1. ESRP1 and common chromosomal deletions. For statistical analysis contingency tables and the chi2-test were performed. Supplementary Figure 2. Validation of ESRP1 and ESRP2 antibodies. ESRP1: Strong staining in ESRP1 overexpressing Hela cells (positive control) and no staining in HeLa wildtype cells (negative control). ESRP2: Strong staining in ESRP2 overexpressing Hela cells (positive control) and no staining in Hela wildtype cells (negative control). [file 12885_2020_7682_MOESM1_ESM.zip › Supplementary materialsR2.docx]

**Supplementary materials**

**Epithelial splicing regulatory protein 1 and 2 (ESRP1 and ESRP2) upregulation predicts poor prognosis in prostate cancer**

Morton Freytag^1^, Martina Kluth^1^, Claudia Hube-Magg^1^, Georgia Makrypidi-Fraune^1^, Hans Heinzer^2^, Doris Höflmayer^1^, Sören Weidemann^1^, Ria Uhlig^1^, Hartwig Huland^2^, Markus Graefen^2^, Christian Bernreuther^1^, Corinna Wittmer^1^, Maria Christina Tsourlakis^1^, Sarah Minner^1^, David Dum^1^, Andrea Hinsch^1^, Andreas Luebke^1^, Ronald Simon^1#^, Guido Sauter^1^, Thorsten Schlomm^3^, Katharina Möller^1^

Supplementary Table 1. ESRP1 and prostate cancer phenotype in ERG subgroups.

Supplementary Table 2. ESRP2 and prostate cancer phenotype in ERG subgroups.

Supplementary Figure 1. ESRP1 and common chromosomal deletions.
